# Non-Destructive Methods and Numerical Analysis Used for Monitoring and Analysis of Fibre Concrete Deformations

**DOI:** 10.3390/ma15207268

**Published:** 2022-10-18

**Authors:** Anna Adamczak-Bugno, Sebastian Lipiec, Martin Vavruš, Peter Koteš

**Affiliations:** 1 Faculty of Civil Engineering and Architecture, Kielce University of Technology, Av. 1000-an. of Polish State 7, 25-314 Kielce, Poland; 2 Faculty of Mechatronics and Mechanical Engineering, Kielce University of Technology, Av. 1000-an. of Polish State 7, 25-314 Kielce, Poland; 3 Faculty of Civil Engineering, University of Žilina, Univerzitná 8215/1, 010-26 Žilina, Slovakia

**Keywords:** fibre concrete, steel fibres, acoustic emission method, numerical analysis, DIC System

## Abstract

The aim of the research was to check the possibility of using the non-destructive method of acoustic emission to assess the condition of concrete without dispersed reinforcement and with various additions of curved steel fibres, during three-point bending. An important aspect of the research proposed in the article is the use of a hybrid method of analysis, which involves complementing the results of strength tests, the results of numerical calculations and the results of strain distributions recorded with a digital image correlation system (DIC System, in this research GOM Suite optical system). The operation of the concrete material under load, depending on the amount of fibres added, is reflected in the recorded acoustic emission (AE) signals. The differences concern the number of signals of individual classes and their distribution over time. The differences exist for both low and high load values, which confirms the possibility of using the acoustic emission method to monitor the condition of the material. It was shown that the numerically determined effective stress levels decreased as the proportion of steel fibres in the concrete increased, while the maximum levels of the first principal stresses increased. During the analyses, a preliminary comparison of the deformation results obtained using the finite element method and the DIC System was also carried out.

## 1. Introduction

### 1.1. Characteristics of Concrete Reinforced with Short, Evenly Distributed Steel Fibres

Steel fibre reinforced concrete (SFRC) is a composite material made of cement, mineral aggregate, sand, water and steel fibres. Unlike conventionally reinforced concrete, it is a homogeneous material with evenly distributed reinforcement. The reinforcement is made of kilometres of fibres in one cubic meter of concrete. The basic parameters of the steel fibres that affect the properties of composite concrete are fibre length, diameter, the tensile strength of the steel used for its production and its geometry. The slenderness ratio, defined as the relation of fibre length to its diameter, was introduced a long time ago. The slenderness ratio of the fibre used as reinforcement should not be less than “50”. Fibre with lower slenderness is always technologically questionable. Another very important feature of the fibre that undeniably affects the characteristics of concrete is its shape. The fibre shape must be selected so that the fibre tips are anchored as securely as possible in the concrete structure [1,2,3].

The primary function of steel fibre in concrete is to reduce stress concentration. When the fibres are evenly distributed in concrete, they transfer loads like bridges through the existing discontinuities of the concrete structure, such as cracks or fractures, taking over the forces resulting from the load. These forces are transferred through the fracture from one side to the other and reduce the stresses at the end of the fracture as a result of the much greater Young’s modulus for steel in relation to the matrix of the surrounding concrete. The described phenomenon prevents the transformation of internal microcracks into larger cracks leading to concrete destruction [4,5].

The main feature of steel fibre reinforced concrete is its resistance to cracking after the appearance of the first structural crack resulting from the load. Contrary to conventional concrete, fibre concrete behaves then not like a brittle material that is subject to destruction but like a flexible object. It is springy and capable to withstand a higher load [5].

The degree of its plasticity, defined in the case of steel fibre reinforced concrete by “fracture resistance”, can be controlled by selecting an appropriate type and amount of fibre. The load–deformation curve shows a very different way of behaviour of concrete composites with steel fibres compared to normal concrete [4,6,7].

There is also a large difference in strength at certain deformations depending on the shape of the fibres. Straight fibres provide worse results than fibres with curved ends, even at higher contents [7].

As a result, concrete that is normally hard but brittle becomes strong and elastic, which translates into many other advantages. An example of this is a fibre concrete floor [8], which thus obtains a flexible structure, limiting the risk of corners and edge chipping. The advantages of steel fibre reinforced concrete result from its higher plasticity and energy absorption capacity. It is especially noticeable in the case of destructive loads at which conventional concrete would fail [3,7,9].

### 1.2. Methods Used in Monitoring and Evaluation of Deformation of Concrete Reinforced with Evenly Distributed Fibres

#### 1.2.1. Acoustic Emission Method

Various research and studies on the use of non-destructive techniques to assess the technical condition of devices and structures operating under high loads have been carried out for at least a dozen years. The method of acoustic emission plays a special role in this respect, as it allows for assessing the overall technical condition of the structure, both in the case of a single load and long-term monitoring of a facility operating under load [10,11,12].

The term acoustic emission (AE) is defined as momentary elastic waves caused by the sudden release of energy stored in the material. These waves are generated in places called AE sources and are usually the result of several phenomena overlapping one another. As a result of an applied external stimulus (e.g., stress, pressure, temperature gradient, magnetic field, etc.), elastic waves propagate from the source to the material boundary surface, where they can be recorded by special receiving transducers [13,14].

In composite materials, which undoubtedly should be considered concrete reinforced with dispersed steel fibres, the basic source of acoustic emission is the formation and development of microcracks. Under load, microcracks can propagate, becoming active sources of AE. Their size and place of occurrence are important factors in the analysis of the threat to the integrity of the structure, as unstable defects usually propagate long before structural failure. In this way, the developing discontinuities become active sources of AE, making it possible to detect emerging hazards [15,16,17].

The values of the parameters of the recorded AE signals allow grouping the signals into classes, each of which is characterised by different dominant destructive processes and a different degree of hazard to the element or the structure. The signals characteristic for each class form “reference” databases enabling the identification of the destruction process, e.g., “concrete crushing” corresponds to the database grouping selected signal parameters assigned to this process [12,17,18,19].

Databases for individual processes (or their groups) are determined using material samples, models in special laboratory tests (where a given destructive process or a group of processes dominates) and full-size structural elements during strength tests and the operation of objects [12,14,15,16].

Having a database of AE reference signals, it is possible in this way to identify active destructive processes occurring in the entire tested volume. By carrying out long-term measurements, it is possible to determine the propagation of damage under the conditions of real load, taking into account the external conditions. An appropriate arrangement of AE sensors allows for the measurement of the entire tested element and the location of the emission source (location of damage) [19,20,21].

NOESIS software is often used to classify reference signals. NOESIS is based mainly on the pattern recognition method in two versions: with an arbitrary division into classes (unsupervised—USPR) and self-learning, in which the division into classes was performed with the use of reference signals (supervised—SPR) [12,16,22,23].

In the first case, the analyses of arbitrary patterns are used mainly to create a database of pattern signals, if the number of classes is unknown. The second method is used when reference signals characterising given destructive processes are available. The reference signals are signals previously collected in databases, generated during independent experiments [18,21,22,23,24].

In the case of statistical methods used to recognise objects, an important issue is the optimal selection of the recorded acoustic emission parameters. Many parameters of the acoustic emission show a strong mutual correlation, which means that they can provide the same information about the AE source. The degree of correlation between the AE parameters is determined by the so-called dendrograms that are created during the grouping of signals with the use of various algorithms depending on the model used [18,21,22,23].

An important issue that can affect the accuracy of the calculations, which should also be taken into account, is the number of iterations needed to obtain satisfactory results. A sufficient number of iterations is 10.000, as proved by experiences in the analysis of concrete elements and structures. Reducing this number caused a significant decrease in signal matching in individual classes while increasing this number slowed down the process of analysis and the obtained results improved the matching to a small extent [19,24,25].

The NOESIS program uses various grouping methods, but the user’s manual of the application does not provide guidelines for selecting one of them. The fuzzy *k-means* algorithm provides accurate results in terms of matching. It belongs to the group of non-hierarchical group algorithms. The algorithm is based on the initial random selection of the location of the centres of the groups. In the subsequent iteration steps, after calculating the function of belonging to individual points to group centres, they are each time recalculated. Such a procedure causes the groups’ centres to search for their correct positions [20,21,22].

In the case of this algorithm, it is obligatory to impose the number of groups. However, the speed of computation and matching make up for these inconveniences.

While the USPR method classifies the AE sources on the basis of the similarity of signals without assigning appropriate mechanisms to the groups, the SPR method assigns specific processes to groups, provided that a database of reference signals is available.

So far, the AE method has been applied to concrete composites reinforced with equally distributed steel reinforcement, primarily to monitor various stages of the cracking process. The analyses focused mainly on the observation of changes related to single parameters. Attempts were made to extend the analyses to the observation of energy and frequency parameters, but as a rule, the values were maintained at the level of 2–3 and were not statistically correlated with each other [21,22,23,24,25,26].

In this paper, the grouping of recorded signals into classes was performed using the *k-means* algorithm. The individual classes were linked to the destructive processes taking place in the material under load. During grouping, 14 signal parameters listed below in this paper were taken into account.

#### 1.2.2. Finite Element Method (FEM)

The developing direction of the loaded concrete behaviour analyses is numerical calculations using the finite element method (FEM) [27,28,29,30,31,32,33]. Abaqus CAE is a computer program that can be used to analyse the parameters of fibre concrete fracture mechanics [34,35]. The results of numerical calculations are the determined material values and parameters: strains, stresses, stress triaxiality coefficient, etc [36]. The essential features of the program ensuring reliable results are: a consistent reflection of the geometry of the analysed element in the program, adoption of boundary conditions to the numerical model, consistent with those observed in experimental tests, appropriate preparation and application of a material relationship, and the development of a high-quality finite element mesh [37]. In the case of numerical analyses related to concrete, the concrete damage plasticity (CDP) [38,39,40,41] model is used when creating the definition of the material model. It requires knowledge of the stress–strain curve obtained through compression and tensile tests of fibre concrete. An important aspect when performing numerical analyses is the ability to verify the obtained results, e.g., by comparing them with experimental results or with the results obtained by real-time laboratory test analysis (use of a video extensometer). 2D and 3D elements are used, depending on the geometry and the forces transferred by the structure. Abaqus program also has a feature of a graphical presentation of the calculation results.

#### 1.2.3. Digital Image Correlation System

The currently available computer programs support the interpretation of the results obtained in the experimental tests of the fracture mechanics parameters. An example is GOM Suite optical analysis software used to analyse the results of non-contact measurements of displacement and deformation of flat and spatial elements subjected to loads [42,43,44].

GOM Suite is a program for preparing shape and dimensional analysis, 3D inspection and processing of meshes from 3D point cloud data and CAD data obtained by fringe projection technique using laser scanners, coordinate measuring machines (CMM) and other measurement systems [45].

GOM Suite software is used, among other things, in the crack analysis of concrete samples, enabling the creation of detailed analyses of crack propagation. It allows us to understand the mechanics of fracture and to observe the directions of complex stresses inside the material.

The problem of failure under load of concrete elements is a current issue being taken up by researchers. Intensive work is underway on the introduction of concrete additives to improve its characteristics, including by using steel fibres or other materials/measures. The number of studies covering the issues of determining the strength characteristics of concrete is associated with the complexity of the destruction process and many factors influencing the process of damage development and final destruction. The source of information about the concrete destruction process is experimental tests carried out in laboratories. Along with the development of new research methods, experimental research is supplemented, among others, by non-destructive testing (acoustic emission, DIC, SEM) [29,46,47,48,49,50,51]. In this work, a hybrid method of analysis was used, consisting of the simultaneous consideration of the results of experimental tests and the accompanying recording of acoustic emission and DIC signals. The applied methodology of analysis was aimed at attempting to determine the parameters characterizing the complexity of the concrete failure process with the addition of steel fibres.

## 2. Materials and Methods

### 2.1. Materials

Three samples with dimensions of 150 × 150 × 700 mm were used in the tests. The samples were different in terms of the steel fibre content. Reference sample *A*_1_ did not contain any fibres. Sample *A*_2_ contained fibres in the amount of 40 kg/m^3^. Sample *A*_3_ contained fibres in the amount of 60 kg/m^3^. The compositions of samples are given in Table 1.

### 2.2. Methods

**Figure 1:**
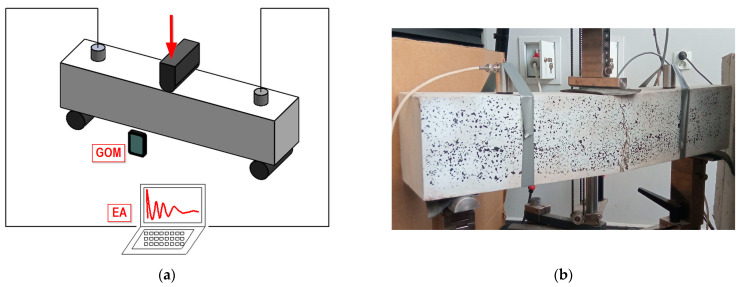
Test stand: (**a**) scheme; (**b**) photograph.

Fibre concrete samples were subjected to three-point bending using a, v (Ulm, Germany). During the tests, the following signals were recorded: test time, traverse displacement and the force loading the specimen. The distance between the supports in the machine was 600 mm. Samples were loaded to destruction. The diagram and view of the test stand are shown in Figure 1.

AE signals were recorded during the tests. For this purpose, an AEWin acoustic emission processor (by Mistras, Physical Acoustics, West Windsor Township, USA) and two VS75-SIC-40dB sensors by Vallen (Wolfratshausen, Germany) were used. Due to the measurement sensitivity of AE sensors, the AE sensors calibration procedure was carried out before the start of target measurements. For this purpose, readings of the AE signal parameters were performed, generated by a reference source—broken graphite of a Pentel pencil with a diameter of 0.3 mm and a hardness of 2H, set at an angle of 30 degrees to the surface of the tested element (Hsu-Nilsen source). The length of the extended graphite was about 2.5 mm. Breaking graphite was associated with the emission of signals with an amplitude above 95 dB. On this basis, it was found that the signals are recorded correctly [12,16,19].

During the study, an image of the front of the sample was recorded for later analysis using a digital image correlation system (in this study using GOM Suite software, ver. 2021, Leipheim, Germany). The use of the DIC System made it possible to determine the level of strain present in the analyzed component. The front of the sample was properly prepared before the test by applying a layer of paint (shown in the photo in Figure 1b).

## 3. Results and Discussion

### 3.1. Strength Tests Results

**Figure 2:**
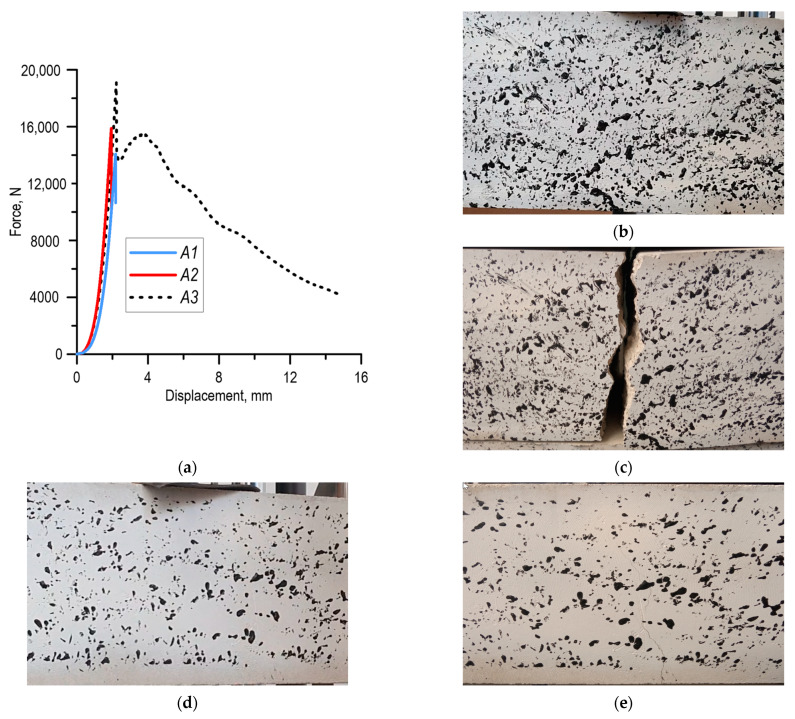

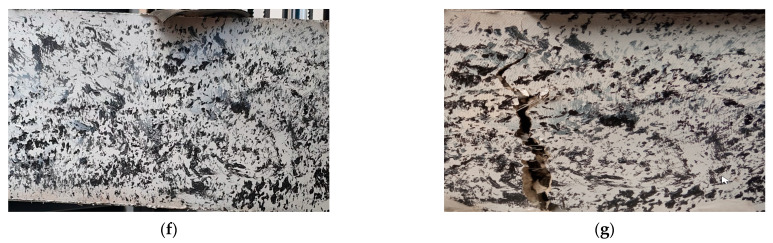
Results of three-point bending tests of analysed specimens: (**a**) force–displacement relationships, view of samples—for small strain: (**b**) *A*_1_, (**d**) *A*_2_, (**f**) *A*_3_, after testing for material: (**c**) *A*_1_, (**e**) *A*_2_, (**g**) *A*_3_.

As a result of the three-point bending tests of fibre concrete samples, force–displacement diagrams were drawn on the strength testing machine. The charts are shown in Figure 2a. The maximal force recorded during the test required to destroy the sample increased with the increase in the amount of steel fibre addition. The elements were loaded by displacement. The lowest force was recorded for the sample without reinforcement, while for the samples with 60% fibre content, the maximum recorded force was higher by about 35%. For the sample with 40% fibre content, the maximum force during the test was higher by approx. 12% in relation to the initial sample (unreinforced) (Table 2). For the fibre concrete specimen without reinforcement and with a 40% share of steel fibre addition, failure during the test occurred shortly after the maximum force was reached. The breakthrough views of the specimens of the three materials analysed after the test are shown in Figure 2b–d. The obtained experimental results became the basis for the implementation of numerical calculations.

### 3.2. The Results of Research That Involved Monitoring of Fibre Concrete with the Use of the Acoustic Emission Method

Fourteen parameters of AE signals were used to create a base of reference signals for destructive processes in the fibre concrete samples:

1. Duration;
2. Rise time;
3. Decay time;
4. RMS;
5. Counts;
6. Counts to peak;
7. Amplitude;
8. Energy;
9. Average frequency;
10. Reverberation frequency;
11. Initiation frequency;
12. Absolute energy;
13. Signal strength;
14. Average Signal Level (ASL).

After dividing the recorded AE signals into four classes, using the *k-means* algorithm, one of the fourteen acoustic emission parameters, which is the signal energy, was used as an illustration of the processes taking place. The individual classes of EA signals, on the basis of preliminary studies, were assigned to the processes taking place in the structure of the tested material:

- Class 1 (blue)—microcrack initiation;
- Class 2 (green)—crack formation and propagation;
- Class 3 (red)—crack development, concrete crushing;
- Class 4 (purple)—plastic deformation, material failure.

**Figure 3:**
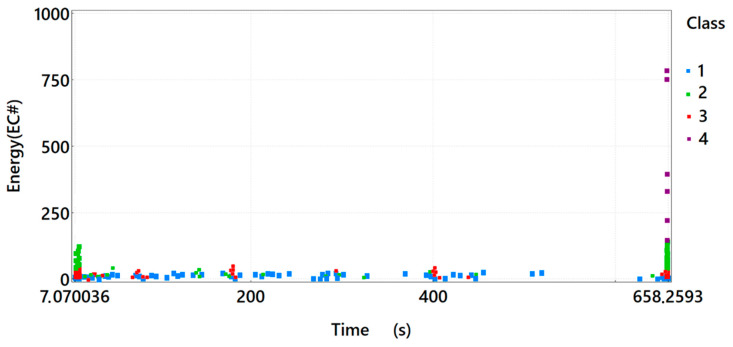
Graph of energy in time, taking into account the division of the recorded signals of acoustic emission into classes for sample *A*_1_.

By analysing the energy distribution of the acoustic emission signals of individual classes in time for sample *A*_1_ (Figure 3), one can observe that the signals of the first three classes appear in the time course from the beginning of the loading process. In particular, the presence of Class 3 signals suggests that at a low level of load and deformation in the tensile zone of the element, damage leading to its destruction occurred. The occurrence of Class 4 signals was equivalent to the destruction of the element.

**Figure 4:**
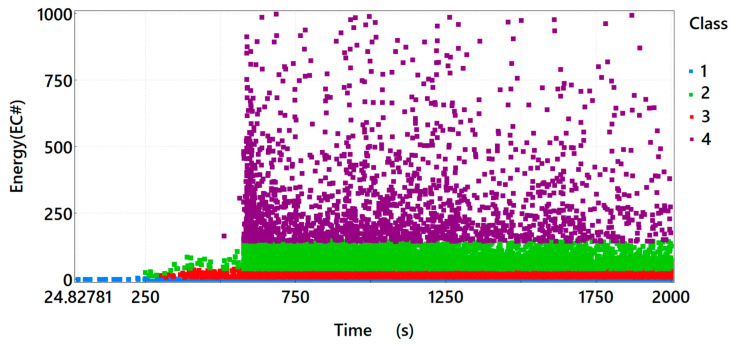
Graph of energy in time, taking into account the division of the recorded signals of acoustic emission into classes for sample *A*_2_.

By analysing the energy distribution of the acoustic emission signals of individual classes in time for Sample *A*_2_ (Figure 4), one can observe that in the time course, in the first phase of the test, Class 1 signals are present, indicating the formation of microcracks in the material under load. Only after 250 s of the test, Class 2 signals related to the formation and propagation of cracks in the tensile zone begin to appear. Soon after the occurrence of Class 2 signals, Class 3 signals also appear, which are related to the development of cracks and the gradual crushing of concrete near the widening crack. Class 4 signals appear after 600 s. Comparing the distribution of Class 4 signals characteristic for Sample *A*_2_ to Sample *A*_1_, one can observe that their number is significantly higher. In the case of the sample reinforced with dispersed steel reinforcement, Class 4 signals indicate material cracking and the formation of the so-called “bridges” associated with taking over the load by the fibres. The sample destruction pattern is not sudden. The loading is accompanied by drops and rises in the force value, while deformation increases.

**Figure 5:**
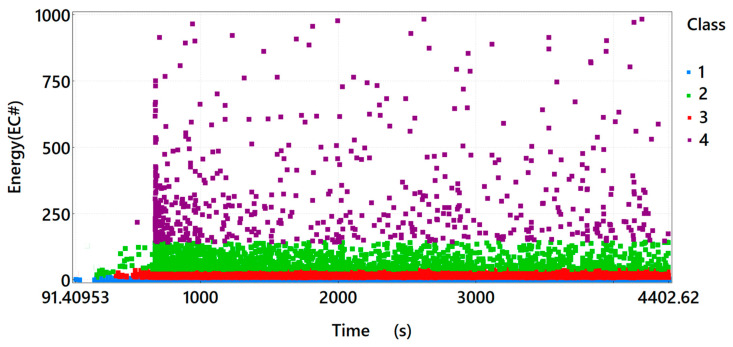
Graph of energy in time, taking into account the division of the recorded signals of acoustic emission into classes for sample *A*_3_.

By analysing the energy distribution of the acoustic emission signals of individual classes in time for sample *A*_3_ (Figure 5), one can observe that in the time course, in the first phase of the test, Class 1 signals are present, indicating the formation of microcracks in the material under load. Only after 300 s of the test, Class 2 signals related to the formation and propagation of cracks in the tensile zone begin to appear. Soon after the occurrence of Class 2 signals, Class 3 signals also appear, which are related to the development of cracks and the gradual crumbling of concrete near the widening crack. Class 4 signals appear after 600 s. Comparing the distribution of Class 4 signals characteristic for Sample *A*_3_ to Sample *A*_1_, one can observe that their number is significantly higher. In the case of the sample reinforced with dispersed steel reinforcement, Class 4 signals indicate material cracking and the formation of the so-called “bridges” associated with taking over the load by the fibres. The sample destruction pattern is not sudden. The loading is accompanied by drops and rises in the force value, while deformation increases.

### 3.3. Results of Numerical Calculations of Fibre Concrete

**Figure 6:**
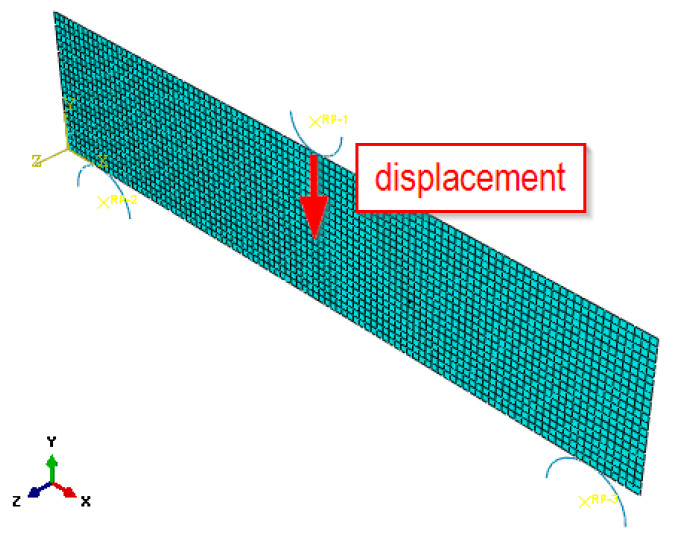
Numerical model of a three-point fibre concrete beam subjected to bending.

Numerical calculations of the fibre concrete element model were carried out using Abaqus software [52]. The three-point bending test of fibre concrete beams was numerically modelled, with different contents of steel fibres. Due to the geometries of the elements analysed in the study, two-dimensional (2D) numerical models were developed. It was assumed that there was no change in stress along the direction of the specimen thickness. This allowed the number of nodes to be reduced and the numerical calculation time to be shortened. The rollers on the supports and the load roller were modelled as rigid structures (Figure 6). A surface-to-surface contact was modelled between the rollers and the beam. In boundary conditions options, the possibility of displacement of the two lower rollers was blocked, while a load in the form of displacement was applied to the upper roller (displacement along the y-axis). The value of the displacement in the numerical calculation program was selected on the basis of the experimental results, which was the displacement recorded at the maximum force during the test. In the numerical model of the beam subjected to bending, 4-node finite elements were used.

An important aspect of performing numerical calculations is the definition of the values present in the fibre concrete damage model, which is the concrete damage plasticity (CDP). Knowledge of the stress–strain curves drawn during the compression and tensile tests of fibre concrete was required. The results found in professional studies served as support in determining the individual parameters of the model [39,53,54,55,56,57]. The individual parameters of the concrete damage plasticity model used in the numerical calculation program are given in Table 3.

**Figure 7:**
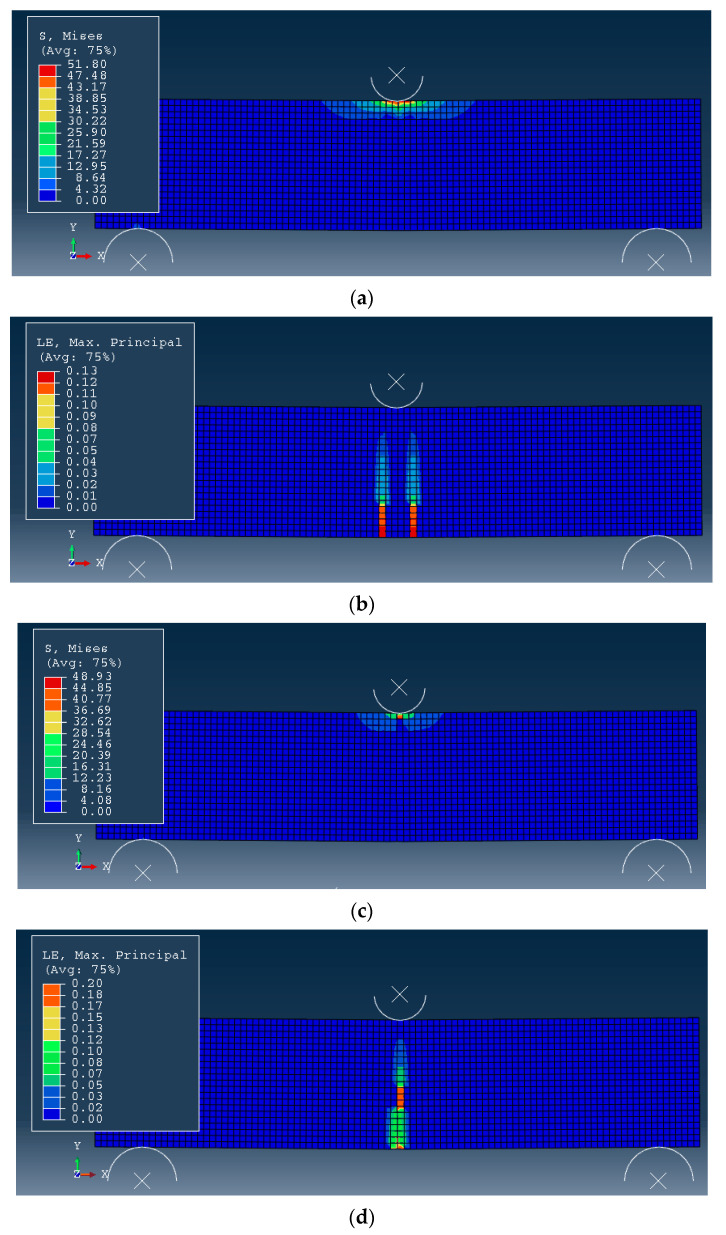

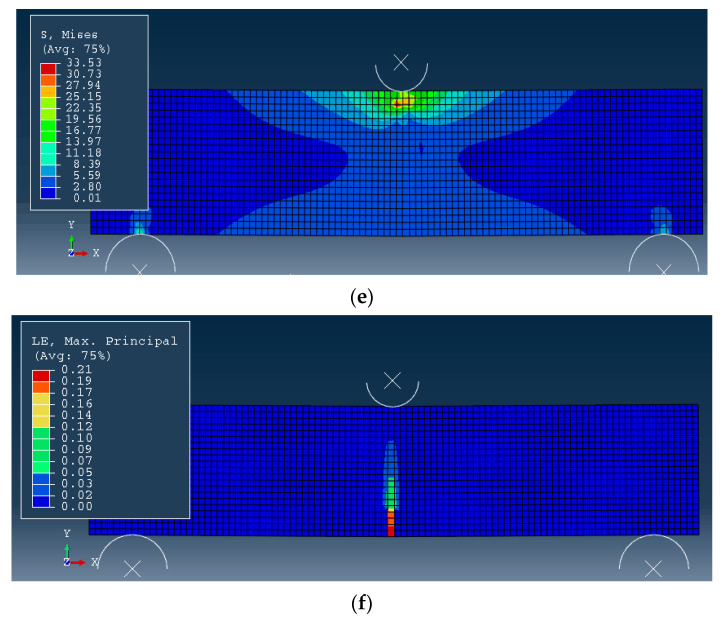
Numerical calculation results of fibre concrete specimens obtained (view from Abaqus program): (**a**) *A*_1_ (effective stresses), (**b**) *A*_1_ (first principal strain), (**c**) *A*_2_ (effective stresses), (**d**) *A*_2_ (first principal strain), (**e**) *A*_3_ (effective stresses), (**f**) *A*_3_ (first principal strain).

As a result of numerical calculations, selected values of the mechanical fields developing in the analysed fibre concrete elements during loading were determined. The results obtained for: effective stress (according to von Mises) and maximum principal strain (first principal strain) are presented (Figure 7, Table 4).

The highest level of effective stress (according to von Mises) occurred for the fibre concrete specimen without the addition of reinforcing steel fibres. As the percentage of steel fibres in the concrete increased (40% and 60%), a decrease in the maximum effective stress level was observed. The decrease in the level of effective stress for the sample without reinforcement and with the proportion of steel fibres (*A*_3_, 60%) was about 35%. In contrast, the highest level of maximum principal strain was recorded for the analysed concrete specimen with steel fibre participation at 60%. It was about 1/3 higher than for the sample without the share of steel fibre reinforcement (*A*_1_).

In the case of the distributions of the fields of maximum principal strains (Figure 7), some differences can be observed between the starting material (*A*_1_) and the other materials analysed (*A*_2_, *A*_3_). The differences took place due to the proportion of steel fibres in the concrete marked *A*_2_ and *A*_3_. The nature of the distribution of effective stresses in the cross-section of the analysed specimens was similar for the three materials included in the numerical analysis.

**Figure 8:**
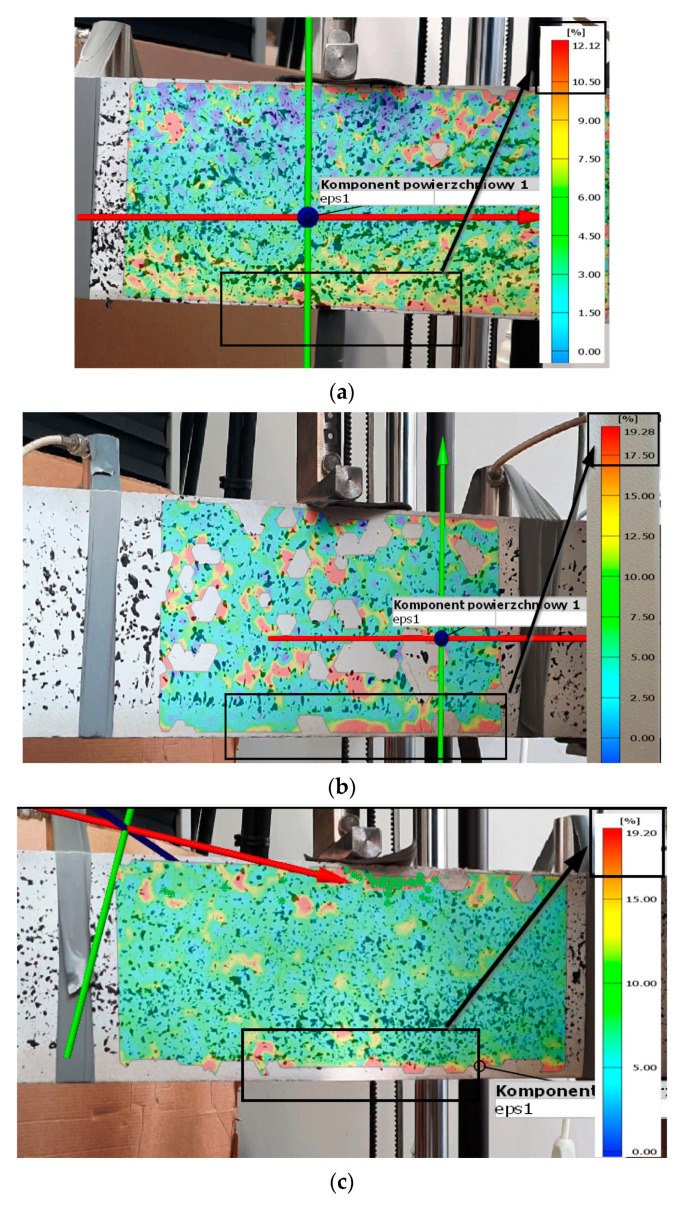
Distributions of the first principal strain determined using GOM software accompanying the experimental tests, view from GOM Correlate software for the sample: (**a**) *A*_1_, (**b**) *A*_2_, (**c**) *A*_3_.

Three-point bending tests on specimens made of the analysed fibre concrete were accompanied by recording the deformation process of the material using the GOM system. In the next step of the analyses, this allowed us to confront the distributions of the first principal strains determined: as a result of numerical calculations and those determined using the GOM Correlate software (Figure 8, Table 5). Within the framework of this work, only the preliminary analyses of the comparison of the two methods of determining the level of deformation in the material will be presented. The maximum difference between the first principal strains determined numerically and as a result of GOM analysis was within 9% (for a material with a steel fibre share of 60%). The smallest difference in deformation results was obtained for the *A*_2_ material of around 3.6%. The results of the GOM system analysis were strongly influenced by the careful application of the paint layer on the sample and the careful positioning of the video recording device during testing in the laboratory.

## 4. Conclusions

The aim of the research was to check the possibility of using the non-destructive method of acoustic emission to assess the condition of concrete without dispersed reinforcement and with various addition of curved steel fibres, during three-point bending. An important aspect of the research proposed in the article was the use of a hybrid method of analysis, which involves complementing the results of strength tests, the results of numerical calculations and the results of strain distributions recorded with the digital image correlation system (DIC System, in this research GOM Suite optical system). Based on the conducted research and analyses, the following conclusions were drawn:

- Three-point bending of fibre concrete samples is associated with the emission of acoustic signals characteristic of various destructive processes occurring in the material.
- The addition of curved steel reinforcing fibres in the form of equally distributed reinforcement changes the number and distribution of acoustic emission signals of individual classes.
- The differences in the number and distribution of acoustic emission signals of individual classes are observed at a low level of load and deformation.
- The acoustic emission method is very useful for monitoring elements or structures made of concrete reinforced with equally distributed steel fibres under load.
- In this paper, a hybrid method of analysis is proposed, which consists in complementing the results of strength tests, the results of numerical calculations and the results of deformation distributions (through the use of the GOM system). The proposed test method allows the determination of the characteristic values of the mechanical fields in the material, the knowledge of which makes it possible to assess the strength of concrete in its initial state and with reinforcement in the form of steel fibre participation.
- It was shown that the numerically determined effective stress levels decreased as the proportion of steel fibres in the concrete increased. However, the maximum levels of the first principal stresses increased (the highest value for specimen material *A*_3_).
- A preliminary comparison of the results of the deformation analyses of the analysed material indicates a fairly good agreement between the results of the distributions of the first principal stresses determined numerically and those obtained from the GOM software analysis. The work with the GOM system requires further experimental research.

Based on the analyses and the conclusions drawn from the research, the authors planned further tests on the monitoring of the condition and deformation of fibre concrete. It is planned to extend the analyses of the recorded acoustic emission signals towards the evaluation of changes in the time–frequency spectra of the recorded acoustic waves. It is also planned to extend the analyses with the use of FEM (introduction of additional parameters, e.g., stress triaxiality factor, Lode parameter) and the GOM system.

## Figures and Tables

**Table 1:** Compositions of samples.

	*A*_1_	*A*_2_	*A*_3_
Concrete mixture	1 kg/m^3^	1 kg/m^3^	1 kg/m^3^
CEM II/B-S 32,5R (Ladce)	400	400	400
Aggregate 0/8 mm	910	910	910
Aggregate 8/16 mm	685	685	685
Fly ash (USS Košice)	80	80	80
Water	200	200	200
Fibres DRAMIX 3D	0	40	60

**Table 2:** Results obtained from three-point bending tests of fibre concrete specimens.

Specimen	Max. Force, N	Displacement (for Max. Force), mm	Max. Displacement, mm
*A*_1_	14,068	2.18	2.18
*A*_2_	15,882	1.94	1.95
*A*_3_	19,088	2.22	14.70

**Table 3:** Details of the CDP material model used in the numerical model.

Material Characteristics/Sample Material	*A*_1_	*A*_2_	*A*_3_
*E,* GPa	37	37	37
ν	0.20	0.2	0.2
Dilation angle	30	30	30
Eccentricity	0.10	0.10	0.10
*f*_b0_/*f*_c0_ (i.e., *σ*_b0_/*σ*_c0_)	1.16	1.16	1.16
*K*	0.67	0.67	0.67
Viscosity parameter	0.00	0.00	0.00
*σ*_c__u,_ MPa	38.72	40.76	42.38
*σ*_t__u,_ MPa	14.07	15.82	19.08

**Table 4:** Maximum values of selected parameters determined by conducting numerical calculations.

Numerical Results/Sample Material	*A*_1_	*A*_2_	*A*_3_
*σ*_eff_ (Mises), MPa	51.80	48.93	33.53
ε_I_, %	13.31	20.01	21.12

**Table 5:** Comparison of the values of the first principal strains determined numerically and those obtained by using the GOM system.

First Principal Strain *ε*_I_, % Determined:	*A*_1_	*A*_2_	*A*_3_
Numerical calculations	13.31	20.01	21.12
GOM system	12.12	19.28	19.20
Differences, %	8.94	3.65	9.09

## Data Availability

Not applicable.
